# An Overview of the Genetic Diversity and Epidemiological Potential of *Yersinia pestis* Populations in Natural Plague Foci of Kazakhstan

**DOI:** 10.3390/pathogens15050551

**Published:** 2026-05-20

**Authors:** Aigul Abdirassilova, Duman Yessimseit, Altynai Kassenova, Altyn Rysbekova, Beck Abdeliyev, Zauresh Zhumadilova, Ziyat Abdel, Raikhan Mussagaliyeva, Tatyana Meka-Mechenko, Galiya Sairambekova, Elmira Begimbayeva, Ainur Nurpeisova, Temirkhan Sagidulin, Ayaulym Maksatova, Sanzhar Agzam, Raikhan Nissanova, Vladimir Motin, Oleg Reva

**Affiliations:** 1 M. Aikimbayev’s National Scientific Center for Especially Dangerous Infections, 14 Zhakhanger St., Almaty A35P0K3, Kazakhstan; yessimseit@gmail.com (D.Y.); kassenovaaltynai@gmail.com (A.K.); rysbekova570@gmail.com (A.R.); abdelbeck@gmail.com (B.A.); zzbgdirect@nscedi.kz (Z.Z.); ziyatabdel@gmail.com (Z.A.); raikhansafar@gmail.com (R.M.); tmekamechenko@gmail.com (T.M.-M.); sairambekovagalya@gmail.com (G.S.); elmira73begimbayeva@gmail.com (E.B.); temirhansagidulin@gmail.com (T.S.); a.maksatova@nscedi.kz (A.M.); sanzhar.dauletuly.agzam@gmail.com (S.A.); 2 Al-Farabi Kazakh National University, 71 al-Farabi Ave., Almaty A15E3B4, Kazakhstan; 3 Kazakh Scientific Research Veterinary Institute, 223 Rayymbek Ave., Almaty A20C2E4, Kazakhstan; nurai1005@gmail.com (A.N.); raihan.nisanova@gmail.com (R.N.); 4 Department of Pathology, The University of Texas Medical Branch, Galveston, TX 77555, USA; vlmotin@gmail.com; 5 Centre for Bioinformatics and Computational Biology, Department of Biochemistry, Genetics and Microbiology, University of Pretoria, Pretoria 0002, South Africa

**Keywords:** *Yersinia pestis*, natural plague foci, Kazakhstan, Central Asia, phenotypic diversity, genotyping, epidemiology, Medievalis, pCKF plasmid

## Abstract

This review provides a comprehensive overview of the genetic diversity and epidemiological potential of *Yersinia pestis* in Kazakhstan’s natural plague foci, emphasizing the link between genotypic variation and outbreak capacity. Integrating historical epidemiological records with contemporary microbiological and genomic data (including PCR, VNTR/MLVA, SNP analysis, and whole-genome sequencing), we evaluate core and accessory genome variations. The data reveal substantial regional heterogeneity. High-risk desert foci (Caspian and Aral regions) are dominated by the Medievalis biovar, including atypical genovariants lacking canonical markers. Conversely, high-mountain foci (Sarydzhaz, Talas) harbor the Antiqua and Talas biovars, primarily linked to enzootic circulation. Notably, the Ili River focus exhibits extreme genomic variability, featuring strains with plesiomorphic traits. Furthermore, the widespread distribution of mobile elements like the cryptic plasmid pCKF suggests significant horizontal transfer contributing to pathogen adaptation. Ultimately, Central Asian plague dynamics are driven by complex evolutionary and ecological interactions. Given climate change and expanding human–wildlife interfaces, continuous genomic and ecological surveillance is essential for the early detection of high-risk *Y. pestis* genovariants and improving public health preparedness.

## 1. Introduction

Plague is a dangerous natural focal infection requiring quarantine that is characterized by extremely high transmissibility and mortality. Three major plague pandemics, which occurred in Late Antiquity (the Plague of Justinian, 6th–8th centuries), the Middle Ages (the Black Death, 14th–17th centuries), and the modern era (the Third Plague Pandemic, 19th–20th centuries), claimed hundreds of millions of human lives [1,2,3,4,5,6].

The causative agent of plague, *Yersinia pestis*, was identified during the third pandemic. It is a Gram-negative, non-motile, non-spore-forming coccobacillus [3]. In nature, it circulates within populations of various rodents (such as gerbils, marmots, ground squirrels, voles, and others) and is transmitted from animal to animal through flea bites [3,7].

**Figure 1:**
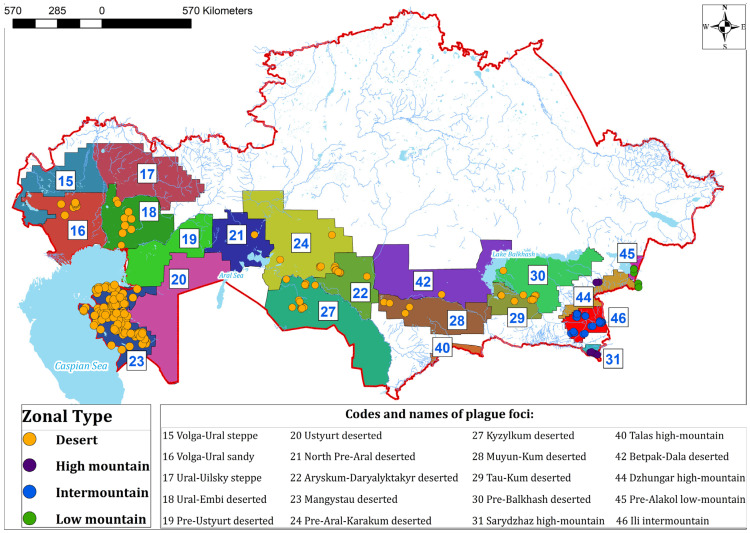
Natural plague foci of Kazakhstan and locations of collection of *Y. pestis* strains deposited in the National Collection of Microorganisms at the M. Aikimbayev’s National Scientific Center for Especially Dangerous Infections, Almaty, Kazakhstan.

Central Asia represents one of the largest and most historically active plague-endemic regions, with Kazakhstan encompassing extensive natural foci that have exhibited sustained epizootic and episodic epidemic activity over the past century [8,9,10] (Figure 1). Recent studies have revealed substantial genetic heterogeneity within *Y. pestis* populations in this region, including the presence of atypical genovariants and mobile genetic elements that may influence pathogen fitness, transmission dynamics, and epidemic potential [11,12]. In parallel, environmental and climatic changes, together with increasing human interactions with wildlife, are likely to alter host–vector relationships and modulate the risk of spillover events [13,14].

This review aims to integrate epidemiological, ecological, phenotypic, and genomic data to provide a comprehensive overview of the diversity and population structure of *Y. pestis* in natural plague foci of Central Asia. The review addresses the hypotheses that (i) distinct *Y. pestis* lineages differ in their epidemic potential according to their ecological and evolutionary background, (ii) regional genetic heterogeneity is shaped by local host–vector systems and environmental conditions, and (iii) accessory genetic elements, including cryptic plasmids, contribute to pathogen diversification and adaptation. Particular emphasis is placed on the emergence of genetically distinct lineages and the implications of genomic diversity for surveillance and public health preparedness under changing environmental conditions. Ultimately, we highlight that the pronounced regional genomic heterogeneity, combined with environmental shifts, may facilitate the emergence of high-risk genovariants, underscoring the critical need for continuous, integrated genomic surveillance.

## 2. Regional Epidemiology and Historical Outbreaks

**Figure 2:**
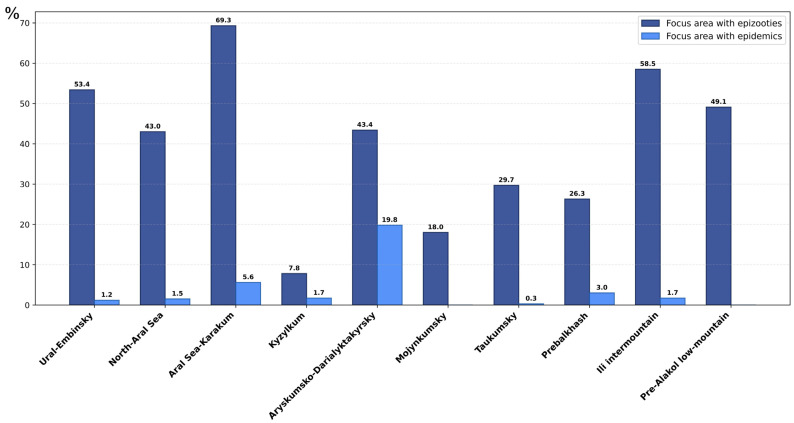
Plague-endemic regions of the Republic of Kazakhstan with high epizootic and epidemic activity. Dark-blue and light-blue bars represent the areas of epizootics and epidemic outbreaks from 1929 to 2021, respectively, estimated as percentages of the total areas of the foci (after Popov et al. [10]).

Long-term surveillance of the circulation of the plague pathogen in wild rodent populations has been conducted in natural plague foci of Kazakhstan since the early 20th century. These studies revealed the highest epizootic activity in plague-endemic areas, especially those located near major natural water bodies. These include the basins of the Caspian Sea, including the Ural–Emba focus, and the Aral Sea, including northern Kyzylkum, Arys-Kum, and Daryalyk-Takyr, as well as Lake Alakol and large active plague foci around Lake Balkhash: Taukum, southern Balkhash, and areas along the middle reaches of the Ili River. Areas of epizootics and epidemic outbreaks from 1929 to 2021, estimated as percentages of the total areas of the foci, are shown in Figure 2 [8,9,10].

**Figure 3:**
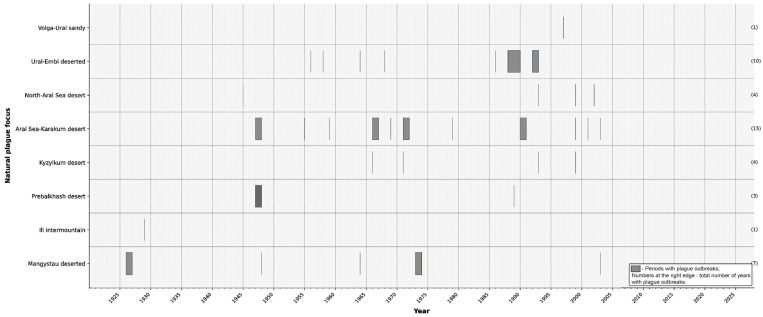
Chronology of human plague outbreaks in different natural foci of Kazakhstan, 1926–2025. Dark blocks indicate periods during which plague outbreaks were recorded in each focus. Sporadic outbreaks are indicated by vertical lines. Numbers in the right margin represent the total number of outbreak years documented for each focus.

Epidemic plague outbreaks are most frequent in the Caspian region, the Aral region, and the southern Balkhash area (Figure 3).

In the Ural–Emba focus, major outbreaks of infection have been documented since 1904–1905, with the most recent outbreak occurring in 1993. These events took place against a background of continuously ongoing widespread epizootics, the activity of which started to decline only by the mid-1990s.

In the Aral region, human plague outbreaks have been recorded since 1924, and from the 1940s onward they occurred almost every decade up to 2003. The high epidemic activity of this region was largely driven by persistent epizootics in the Aral Karakum area, where most plague outbreaks occurred. In the remaining enzootic territories of the Aral region, including the North Caspian focus and the northern part of the Kyzylkum Desert, the epizootic process was characterized by periodic activity. In this region, plague epidemics were first identified in 1945 following a high-mortality outbreak in desert areas of the Northern Aral region, where it still persists now [8,9,10].

In southern Balkhash and the Ili River valley, epidemic years were recorded far less frequently than in the previously described regions. Two major outbreaks happened in 1929 in the left bank of the middle Ili River, and in 1947–1948 in the southern shore of Lake Balkhash. These outbreaks were clearly anthropogenic in origin. They were characterized by rapid transmission across multiple settlements and exceptionally high mortality rates (95% and >70%, respectively), driven by a high proportion of primary and secondary pneumonic plague cases [8,9,15].

Human infections typically occurred during periods of intensified epizootic activity and were confined to enzootic territories. Over the entire observation period starting from the early 20th century to the present, the Central Asian desert plague focus recorded 2280 human cases, including 2066 fatalities (case-fatality rate: 90.6%). Of these, 37% of cases were registered in Kazakhstan, with a mortality rate of 80.0% [8,9]. According to the publication by Sagiyev et al. (2019) [16], annual vaccination with EV76 NIIEG has been performed since the mid-1930s among residents of plague-enzootic regions. Vaccination campaigns were generally conducted in spring before peak epizootic activity. Plague vaccination is still performed in modern Kazakhstan, although on a smaller and more targeted scale than during the USSR period. Changes in vaccination strategy may potentially influence the phenotypic and genotypic structure of pathogen population [17,18]; however, studies addressing this issue in *Y. pestis* remain limited. Interactions between the pathogen and its natural rodent hosts may also contribute to shaping population variability. Wild rodent populations inhabiting endemic plague foci often contain animals with detectable anti-*Y. pestis* antibodies, indicating prior exposure and survival. In addition, experimental and field serological studies conducted in Madagascar and other endemic regions have demonstrated measurable humoral immune responses in wild rodents following natural exposure [19].

## 3. Bacteriological and Modern Genetic Approaches Used to Study Diversity of *Y. pestis* Isolates

The study of the phenotypic and genetic diversity of *Y. pestis* has undergone a substantial transition from classical microbiological characterization to integrative, genome-scale approaches. Conventionally, strain differentiation relied on phenotypic assays, including biochemical profiling, assessment of virulence-associated traits such as fraction I (F1 antigen) production, sensitivity to bacteriophages and pesticin, and evaluation of nutritional requirements. These methods remain important for distinguishing biovars and identifying atypical variants, particularly in field and surveillance settings, although their discriminatory power is limited and often influenced by environmental conditions [4,20].

The introduction of molecular methods has significantly improved resolution in strain typing. Targeted PCR assays have been widely used to detect diagnostic polymorphisms in genes such as *rpoB*, *glpD*, *napA*, *araC*, *ssuA*, and *rhaS*. These approaches provide rapid and specific identification of major biovars and clades, facilitating routine surveillance and outbreak investigations [4,21,22]. However, their ability to resolve fine-scale evolutionary relationships is inherently limited.

To overcome these constraints, multilocus variable-number tandem repeat analysis (MLVA/VNTR typing) has been extensively applied in studies on *Y. pestis* strains [23,24,25]. By exploiting variation in tandem repeat loci, VNTR-based approaches generate high-resolution genotypic profiles that are particularly useful for epidemiological tracking and investigation of transmission chains. Nevertheless, the relatively high mutation rate of repeat regions compared to the core genome regions can introduce homoplasy, reducing phylogenetic robustness in long-term evolutionary analyses [11].

Single-nucleotide polymorphism (SNP)-based genotyping has provided a more stable framework for reconstructing evolutionary relationships. SNPs represent the most abundant and phylogenetically informative form of genetic variation, enabling robust delineation of major lineages, sublineages, and clades of *Y. pestis*. In combination with phylogenetic inference methods, SNP analysis has greatly enhanced our understanding of the global spread and diversification of the pathogen. Whole-genome sequencing (WGS) now represents the central approach for studying *Y. pestis* diversity. WGS provides comprehensive resolution of genetic variation, including SNPs, insertions and deletions, structural rearrangements, and the presence of mobile genetic elements. Comparative genomic analyses based on WGS data have revealed substantial heterogeneity within natural populations, including the existence of atypical genovariants and region-specific adaptations. Importantly, genome-scale data allow for the reconstruction of transmission pathways, identification of evolutionary bottlenecks, and assessment of population structure at an unprecedented resolution [11,12].

In addition to core genome variation, increasing attention has been directed toward the role of mobile genetic elements, including plasmids, transposons, and CRISPR-Cas systems. The analysis of CRISPR spacer arrays provides insight into historical interactions with bacteriophages and other mobile elements, offering an additional layer of resolution for distinguishing closely related strains. Similarly, the detection of accessory elements, such as cryptic plasmids, highlights the importance of horizontal gene transfer in shaping the genetic landscape of *Y. pestis* populations [26,27].

Recent advances have further expanded the analytical framework through the integration of multi-omics approaches. Transcriptomic, proteomic, metabolomic, and epigenetic studies enable the functional characterization of genetic variation, linking genotype to phenotype under different environmental and host-associated conditions. These approaches provide a systems-level understanding of pathogen adaptation, virulence regulation, and host–vector interactions, complementing static genomic analyses [12,28,29,30].

Finally, modern phylogenomic and population genomic frameworks integrate large-scale datasets using robust computational methods, including parsimony, maximum likelihood, and Bayesian inference. These approaches enable the identification of clonal expansions, reconstruction of evolutionary trajectories, and correlation of genetic variation with ecological and epidemiological factors. Together, they provide a comprehensive toolkit for monitoring the emergence and spread of *Y. pestis* lineages in natural foci and for informing public health interventions [31].

It can be concluded that the study of *Y. pestis* diversity has evolved into a multidisciplinary field that combines classical microbiology with advanced genomic and systems biology approaches. This integrative strategy is essential for understanding the evolutionary dynamics of the pathogen, identifying emerging high-risk variants, and improving surveillance in plague-endemic regions.

## 4. Subspecies and Phenotypic Diversity

In Kazakhstan, plague enzooticity is maintained by two subspecies of *Yersinia pestis*: *Y. pestis* subsp. *pestis* belonging to biovars Antiqua (ANT) and Medievalis (MED) and *Y. pestis* subsp. *central asiatica* (Talas biovar, 0.PE4t). Strains belonging to the Talas biovar are confined to the transboundary Talas Mountain plague focus, the majority of which lies within Kyrgyzstan. Although most isolates recovered within Kazakhstan exhibit phenotypic and genotypic characteristics consistent with their respective taxonomic groups, atypical strains deviating from canonical profiles are not uncommon [32,33].

Microbiological and molecular genetic studies of a large number of strains have demonstrated that *Y. pestis* of the MED circulates in the plague foci of the Caspian and Aral Sea regions [11,34,35].

The strains of the MED biovar bears several mutations common with other ‘modern’ biovars of *Y. pestis* (e.g., Antiqua (ANT)). These mutations align with established evolutionary trajectories from *Yersinia pseudotuberculosis* to highly virulent *Y. pestis*. Key adaptations include a frameshift mutation in the Rcs regulatory pathway and dysregulation of cyclic di-GMP signaling, leading to the activation of the *hms* locus and biofilm-mediated blockage of the flea foregut. This blockage promotes repeated feeding attempts by infected fleas, substantially enhancing transmission efficiency to mammalian hosts, including humans. Additionally, inactivation of *ureD* suppresses urease activity and is thought to have undergone strong positive selection, further optimizing flea-borne transmission [36,37,38,39].

Phenotypic diversity between *Y. pestis* strains isolated from different geographical areas was observed. For example, the strains isolated in the Ural–Emba focus exhibit variability in sensitivity to pesticin 1 and require nutrient supplementation with methionine, cysteine, and threonine for growth in culture media. Strains circulating in the Aral region largely represent typical members of the main subspecies; however, isolates from the Kyzylkum autonomous focus display amino acid auxotrophy, with growth dependent on leucine, arginine, lysine, and cysteine. These populations include F1-deficient strains with reduced virulence in laboratory mice and delayed rhamnose fermentation taking 2 to 13 days. Strains from the Balkhash region and the Ili River valley show pronounced phenotypic heterogeneity. In addition to canonical Central Asian desert focus strains, the Balkhash autonomous focus harbors variants with altered traits, including reduced FI synthesis, resistance to plague bacteriophage, and inability to ferment maltose. Amino acid-dependent strains (arginine and tryptophan auxotrophy) with reduced and host-selective virulence are also observed in this region. Notably, arabinose-negative (Ar–) atypical strains have persisted in this region and have even expanded their geographic range. Within the Ili River intermountain autonomous focus, isolates exhibit heterogeneity in sensitivity to pesticin 1 and bacteriophage L-413C, and occasional strains with weak denitrification activity have been reported [8,10,20].

## 5. Molecular Typing and Genomic Analysis

In recent previous publications, more than 100 *Y. pestis* natural isolates have been characterized by phenotype and genotype [11,12]. This work continues and here we present results of a bacteriological analysis and genotyping of 17 new *Y. pestis* strains representing distinct natural plague foci of Kazakhstan. The on-going study is aimed at identifying potential associations between genotypic variation and the epidemic potential of different genetic variants of the pathogen in Kazakhstan.

The analyzed strains exhibited phenotypic characteristics consistent with *Y. pestis*, including susceptibility to both species-specific and broad-host-range bacteriophages, confirming their taxonomic identity. VNTR and SNP typing of 17 representative isolates further supported their assignment to the MED biovar, although a subset of the strains contained plasmid pCKF and displayed divergent genotypic profiles at med24 locus (Table 1).

The genetic diversity of the selected strains of *Y. pestis* isolates has been revealed using PCR-based assays and whole-genome sequencing (WGS). Polymorphisms in the *napA*, *araC*, *ssuA*, and *rhaS* genes together with transposon insertions and CRISPR-Cas spacer patterns were used.

PCR amplification targeting the variable region of *rpoB* detected no deletions characteristic of the Orientalis biovar in these isolates, and the primers targeting the variable region of *glpD* revealed no polymorphism among the strains. The MED-specific primer sets (med24 and ANT/MED) classified the majority of isolates within the MED clade of *Y. pestis*. Canonical Medievalis strains typically harbor a 24 bp deletion in the region flanked by Med24 primers, distinguishing them from Antiqua and non-mainline lineages (e.g., Talas), which lack this deletion.

**Figure 4:**
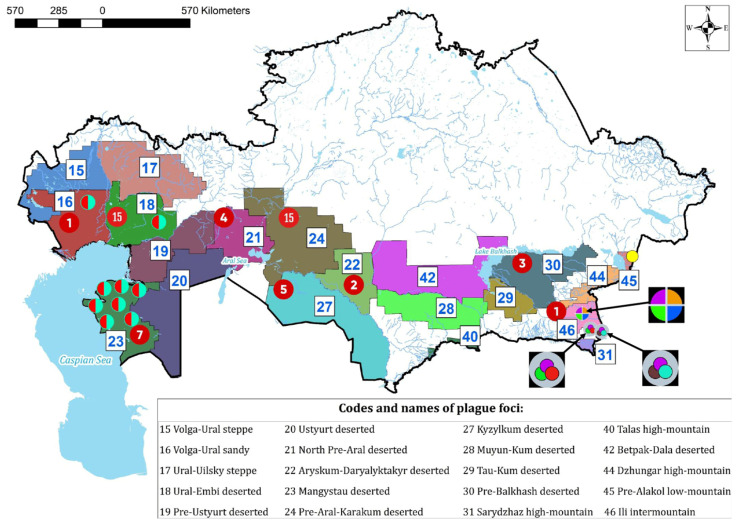
Distribution of *Yersinia pestis* genovariants across plague foci of the Republic of Kazakhstan. The map indicates key genetic and phenotypic features of isolates, including absence of the 24 -bp deletion in the Med24-flanked DNA region (red sectors); presence of the cryptic plasmid pCKF (dark blue sectors); and insertion of CRISPR-Cas elements similar to the large genomic island found in *Y. pseudotuberculosis* (pink sectors). Additional markers include insertion of a transposase and a large hypothetical gene within the *yhfZ* locus (yellow sectors); absence of the 5′-end deletion in the *ilvN* gene (green sectors); a nucleotide substitution (A → G) at position 671 in *rhaS* characteristic of *Y. pseudotuberculosis* and non-main *Y. pestis* (orange sectors); absence of the G → T substitution in the *napA* gene, indicating a functional periplasmic nitrate reductase (den^+^ phenotype, light blue sectors); absence of the *yeaW* deletion in the 2.ANT/2.MED region (turquoise sectors); and retention of both *yapB1* and *yapB2* genes due to lack of the typical deletion (dark brown sectors). The map further denotes the number of recorded outbreaks (red circles with numbers in the middle) in each focus and identifies strains carrying multiple mutations as sectors of pie diagrams. The diagram summarizes the results of genotyping of *Y. pestis* strains from the National Collection of Microorganisms (NCMO) at the M. Aikimbayev’s National Scientific Center for Especially Dangerous Infections (NSCEDI) in Almaty, Kazakhstan. Whole-genome sequences of these strains are available through BioProject PRJNA1249055 at NCBI.

Unexpectedly, isolates from seven Caspian-region foci, although assigned to the MED biovar, lacked the characteristic 24 bp deletion in this locus (Figure 4). The biological significance of this genovariant remains unclear and warrants further investigation. Notably, as explained above, the regions where these strains were identified have historically experienced intense epidemics with high mortality.

## 6. Genetic Diversity of *Y. pestis* Isolates from Different Plague Foci of Kazakhstan

### 6.1. Ili River Focus

The Ili River intermountain plague focus harbors the most genomically diverse population of *Y. pestis* (Figure 4). Among the 30 analyzed strains, 8 exhibited structural variation in chromosomal sequences, with 3 strains showing alterations across multiple loci. Several isolated strains shared plesiomorphic features resembling *Y. pseudotuberculosis* isolates. Usually, members of the main “modern” clade of *Y. pestis* can be distinguished from *Y. pseudotuberculosis* and ancestral *Y. pestis* lineages through an analysis of the acetolactate synthase small subunit gene (*ilvN*) [40,41]. Notably, *ilvN* sequences from two isolates originating from the Ili River intermountain focus in the southern Balkhash region showed similarity to the *Y. pseudotuberculosis* reference strain, lacking the 45 bp deletion at the 3′ end of the gene. In contrast, canonical representatives of the main *Y. pestis* clade harbor a truncated *ilvN* gene, which is shorter by 45 nucleotides.

Several other marker strains listed below were isolated in this region.

Strain 37_YP92_S3, isolated from *Rhombomys opimus* on 26 June 2007, lacked the 5′-end deletion in *ilvN*; at position 671 of *rhaS*, it carried a G instead of A, consistent with *Y. pseudotuberculosis* and non-main *Y. pestis* biovars. It also displayed a distinct *napA* structure, lacking the G substitution at position 613, implying a functional periplasmic nitrate reductase and denitrification-positive (den^+^) phenotype. Additionally, its genome contained CRISPR-Cas insertions corresponding to the large genomic island (GI) typical of *Y. pseudotuberculosis* and ancestral *Y. pestis*, but with mutations in the target region recognized by the PCR analysis.

Strain 43_YP23_S22 isolated from *R. opimus* (7 July 2010) similarly lacked the 5′ *ilvN* deletion and harbored CRISPR-Cas GI insertions analogous to those in *Y. pseudotuberculosis* and non-main *Y. pestis*, but without the primer-target mutations observed in 37_YP92_S3. Moreover, it lacked the characteristic 24 bp deletion in the region flanked by Med24 primers, deviating from canonical MED strains.

Strain 19_S74 isolated from *Xenopsylla gerbilli* (15 June 2022) showed no *yeaW* deletion in the 2.ANT/2.MED region as in *Y. pseudotuberculosis* and *Y. pestis* Antiqua (ANT) strains. It carries the long CRISPR-Cas insert, and uniquely retained intact *yapB1* and *yapB2* genes, as in *Y. pseudotuberculosis*. In contrast, canonical *Y. pestis* strains harbor a fused *yapB* gene resulting from a deletion spanning the 3′ portion of *yapB1* and the 5′ portion of *yapB2*.

An additional five strains from this focus also lacked the 24 bp deletion in the Med24-flanked region.

It should be noted that the Ili River intermountain focus is characterized by persistent epizootic activity. A representative case involved a man who acquired infection while hunting a wild cat; he developed septicemic and secondary pneumonic plague and died on the third day. The outbreak was clearly anthropogenic in origin [9,15]. The genetic diversity of the *Y. pestis* population including atypical strains exhibiting plesiomorphic features, together with ongoing climatic changes, may facilitate the emergence of high-risk genovariants. Historically, the region experienced substantial plague morbidity, with only major outbreaks formally documented, including the 1929–1930 Kosagach outbreak in the left-bank Ili Basin [9,15].

### 6.2. Caspian Region Population Structure

Another region exhibiting pronounced genomic structural variability in *Y. pestis* populations is the Caspian region, which encompasses four natural plague foci: the Volga–Ural sandy, Ural–Emba, Pre-Ustyurt, and Mangystau autonomous desert foci. The Mangystau focus, defined by a unique ecological landscape and a long history of plague persistence, requires sustained and rigorous epidemiological surveillance to mitigate outbreak risk and ensure public health security [42]. Our data indicate the presence of atypical strains in this region combining carriage of the small plasmid pCKF with the absence of the characteristic 24 bp deletion in the Med24-flanked locus that is a hallmark of these strains. These variants were predominantly associated with the Mangyshlak focus, suggesting localized genetic divergence within the Medievalis lineage [12].

Isolates from the Mangyshlak focus formed a compact, genetically homogeneous cluster, whereas strains from the Volga–Ural, Pre-Ustyurt, and Ural–Emba foci partial overlap, reflecting historical epidemiological connectivity. Notably, the small plasmid pCKF detected in eight isolates spanning all four Caspian foci (Table 1), showed complete identity to pCKF plasmids previously reported in Caucasian strains of biovar MED0 [33,43], consistent with horizontal transfer and/or long-term circulation across geographically separated regions. Despite the sequence identity of pCKF plasmids from the Caucasus and Kazakhstan, their host strains differ substantially, belonging to distinct biovars (MED1 instead of MED0). This suggests that pCKF can circulate independently of the bacterial genomic background across geographically separated regions. These findings, together with the occurrence of atypical strains lacking the Med24 24 bp deletion, highlight the need for continuous genomic surveillance to detect emergent virulent or drug-resistant clones and to clarify the role of pCKF in virulence and environmental persistence [11,12].

Historically, the Caspian region exhibited high epidemic activity against a background of acute epizootics. In the Volga–Ural sandy focus, outbreaks were recorded in 1899, 1904–1907, 1909–1918, 1922–1930, 1932–1933, 1935–1941, 1945–1946, 1951, 1975, and 1997, totaling 38 epidemic years (117 events). Transmission was frequently associated with synanthropic rodents (~15%), including flea-borne infection via *Pulex irritans*; handling of infected camels accounted for ~10% of outbreaks, with the last such event in the Atyrau Region in 1975. The most recent case in this focus occurred in 1997 during a large-scale epizootic outbreak (1997–1998), and 19 outbreaks were secondary, driven by human-to-human spread [9,44].

Persistent activity in the Ural–Emba focus led to recurrent outbreaks. Thus, between 1938 and 1993, nine epidemic foci with 12 cases were documented, predominantly linked to flea bites; a single camel-associated outbreak occurred in 1958 (4 cases). The last cases were recorded in 1990 (fatal) and 1993 in the Atyrau Region [34,35]. In the Pre-Ustyurt focus (identified in 1957), seven epidemic foci with 11 cases were reported by 1997, which were largely sporadic (1958, 1959, 1961, 1968, 1988), with one cluster in August 1967 (5 cases) associated with the slaughter of an infected camel [4,5,6,34]. In the Mangyshlak focus, 22 outbreaks and 58 cases were recorded between 1926 and 1974, with infection sources including camels (18.2%), game animals (9%), and flea bites (72.8%) [9,45].

As in other plague foci, transmission was predominantly vector-borne via bites of infected fleas. Most human cases occurred among livestock workers and their families, with bubonic plague predominating (63.8%), followed by pneumonic (17.2%) and septicemic forms (15.6%); cutaneous–bubonic cases accounted for 3.4% [45].

### 6.3. Sarydzhaz and Talas Mountain Foci

Isolates from the Sarydzhaz and Talas Mountain foci, which belonged either to ANT or to Talas biovars, produced longer PCR amplicons with these primers compared to those characteristic of MED isolates [11]. There is no evidence that ANT or Talas strains from the Sarydzhaz and Talas high-mountain foci have been directly associated with documented human epidemic outbreaks. They are primarily linked to enzootic circulation in wildlife reservoirs, with only indirect or uncertain links to human disease.

## 7. Conclusions

The present review highlights the exceptional complexity of *Yersinia pestis* populations circulating within the natural plague foci of Kazakhstan, where long-term enzootic persistence is coupled with episodic epidemic manifestations. The integration of historical epidemiological data with modern genomic approaches demonstrates that plague dynamics in Central Asia are driven by a combination of ecological, evolutionary, and anthropogenic factors. Extensive surveillance conducted over more than a century has revealed that the highest epidemic activity is associated with lowland desert foci of the Caspian and Aral regions, whereas mountain foci such as Sarydzhaz and Talas function primarily as reservoirs of ancient lineages with limited epidemic potential [8,9,10,40].

A key finding emerging from recent studies is the pronounced genetic heterogeneity of *Y. pestis* populations in Kazakhstan, particularly within the Ili River intermountain and Caspian regions. The identification of atypical genovariants, including strains lacking canonical deletions or retaining plesiomorphic features characteristic of *Y. pseudotuberculosis*, indicates ongoing microevolutionary processes within natural foci. These observations are consistent with the hypothesis that Central Asia represents a major evolutionary reservoir of *Y. pestis*, where diversification is shaped by an intensive introgression of genetic variants of the pathogen across geographically distant plague foci, creating admixed populations containing 5 to 45% introgressed genomic component [11], as well as by adaptation to specific host–vector systems and environmental conditions [12,35].

The application of modern molecular approaches, including WGS, SNP-based phylogenetics, and analysis of mobile genetic elements, has significantly improved our ability to resolve population structure and trace evolutionary trajectories. In particular, the detection of the small plasmid pCKF across geographically distant foci and distinct genetic backgrounds suggests that horizontal gene transfer plays a previously underappreciated role in shaping the accessory genome of *Y. pestis*. Although the functional significance of such elements remains unclear, their distribution raises important questions regarding their potential contribution to virulence, environmental persistence, and transmission efficiency [34,43].

At the same time, phenotypic variability observed among isolates, including differences in metabolic requirements, virulence traits, and bacteriophage sensitivity, underscores the importance of integrating classical microbiological methods with genomic analyses. Such combined approaches are essential for the accurate characterization of strains and for linking genotype to epidemiologically relevant phenotypes, particularly in the context of surveillance and outbreak investigation [21,22].

Importantly, the data reviewed here indicate that not all *Y. pestis* lineages contribute equally to epidemic risk. While strains of the MED biovar dominate in historically active epidemic regions, ANT and Talas lineages circulating in high-mountain foci appear to be largely restricted to enzootic cycles and are not clearly associated with major human outbreaks. This distinction highlights the need to consider lineage-specific ecological and evolutionary characteristics when assessing plague risk [40,41].

Looking forward, the continued expansion of plague foci, now covering approximately 41% of the territory of Kazakhstan, combined with ongoing climate change and increasing human–wildlife interactions, is likely to alter host–vector dynamics and create new conditions for pathogen emergence. These changes may facilitate the appearance of novel genovariants with enhanced epidemic potential, particularly in regions where genetically diverse populations are already present. In this context, continuous genomic surveillance, integrated with ecological monitoring, epidemiological analysis, and public health surveillance, are essential for the early detection of emerging variants and for the development of effective prevention strategies. Future research should focus on elucidating the functional significance of genetic variation, including the role of accessory genome elements, and on understanding how environmental and host-related factors influence the evolution and transmission of *Y. pestis*. Such multidisciplinary efforts will be critical for improving preparedness and mitigating the risk of future plague outbreaks in Central Asia and beyond.

## Figures and Tables

**Table 1:** Results of biochemical assays, phage typing, and PCR analysis of 17 recent representative MED isolates.

Isolate ID	Phage Susceptibility (*Y. pestis*)	Phage Susceptibility (*Y. pseudotuberculosis*)	Glycerol	Rhamnose	Arabinose	Nitrate Reduction	*glpD* (bp)	*med24* (bp)	ANT/MED (bp)	pCKF (bp)
14_YP67_VU	+	+	+	−	+	−	508	222	397	472
15_YP19_VU	+	+	+	−	+	−	508	222	397	472
13_YP18_MANG	+	+	+	−	+	−	508	222	397	472
11_YP48_VU	+	+	+	−	+	−	508	222	397	472
10_YP11_Atyr	+	+	+	−	+	−	508	198	397	0
12_YP00_VU	+	+	+	−	+	−	508	222	397	472
9_YP22_Akt	+	+	+	−	+	−	508	222	397	472
13_YP09_MANG	+	+	+	−	+	−	508	222	397	472
10_YP48_VU	+	+	+	−	+	−	508	198	397	0
16_YP04_PKK	+	+	+	−	+	−	508	198	397	0
27_YP43_PKK	+	+	+	−	+	−	508	198	397	0
15_YP93_NP	+	+	+	−	+	−	508	198	397	0
11_YP43_NP	+	+	+	−	+	−	508	198	397	0
7_YP90_NP	+	+	+	−	+	−	508	198	397	0
9_YP24_NP	+	+	+	−	+	−	508	198	397	0
9_YP1_NP	+	+	+	−	+	−	508	198	397	0
27_YP6_NP	+	+	+	−	+	−	508	198	397	0

Diagnostic reactions are indicated as positive (+) or negative (−), and the lengths of diagnostic PCR products are shown in base pairs (bp).

## Data Availability

Genome sequences discussed in this review are available through BioProject PRJNA1249055 at NCBI.

## Abbreviations

The following abbreviations are used in this manuscript:

ANT	Antiqua
MED	Medievalis
PCR	Polymerase Chain Reaction
SNP	Single-Nucleotide Polymorphism
MLVA	Multiple-Locus Variable-Number Tandem Repeat Analysis
VNTR	Variable Number of Tandem Repeat
CRISPR	Clustered Regularly Interspaced Short Palindromic Repeat
WGS	Whole-Genome Sequencing
